# Non-Destructive Quality Evaluation of Pepper (*Capsicum annuum L.*) Seeds Using LED-Induced Hyperspectral Reflectance Imaging

**DOI:** 10.3390/s140407489

**Published:** 2014-04-24

**Authors:** Changyeun Mo, Giyoung Kim, Kangjin Lee, Moon S. Kim, Byoung-Kwan Cho, Jongguk Lim, Sukwon Kang

**Affiliations:** 1 National Academy of Agricultural Science, Rural Development Administration, 150 Suinro, Gwonseon-gu, Suwon, Gyeonggi-do 441-100, Korea; E-Mails: cymoh100@korea.kr (C.M.); gadinlee@korea.kr (K.L.); limjg@korea.kr (J.L.); skang@korea.kr (S.K.); 2 Environmental Microbiology and Food Safety Laboratory, BARC-East, Agricultural Research Service, US Department of Agriculture, 10300 Baltimore Avenue Beltsville, MD 20705, USA; E-Mail: Moon.Kim@ars.usda.gov; 3 Department of Biosystems Machinery Engineering, College of Agricultural and Life Science, Chungnam National University, 99 Daehak-ro, Yuseong-gu, Daejeon 305-764, Korea; E-Mail: chobk@cnu.ac.kr

**Keywords:** hyperspectral imaging, pepper seed, quality, PLS-DA, LED

## Abstract

In this study, we developed a viability evaluation method for pepper (*Capsicum annuum L.*) seeds based on hyperspectral reflectance imaging. The reflectance spectra of pepper seeds in the 400–700 nm range are collected from hyperspectral reflectance images obtained using blue, green, and red LED illumination. A partial least squares–discriminant analysis (PLS-DA) model is developed to classify viable and non-viable seeds. Four spectral ranges generated with four types of LEDs (blue, green, red, and RGB), which were pretreated using various methods, are investigated to develop the classification models. The optimal PLS-DA model based on the standard normal variate for RGB LED illumination (400–700 nm) yields discrimination accuracies of 96.7% and 99.4% for viable seeds and nonviable seeds, respectively. The use of images based on the PLS-DA model with the first-order derivative of a 31.5-nm gap for red LED illumination (600–700 nm) yields 100% discrimination accuracy for both viable and nonviable seeds. The results indicate that a hyperspectral imaging technique based on LED light can be potentially applied to high-quality pepper seed sorting.

## 1. Introduction

Seeds are one of the most fundamental elements of agriculture and the value of the global seed market reached $7.8 billion in 2011. Peppers (*Capsicum annuum L.*) are a typical spicy seasoning and one of the most popular vegetables in South Korea. Peppers are used as the raw materials in a variety of foods as seasonings, pepper paste, or kimchi. The selection of healthy seeds with high germination rates is important for ensuring a yield of high quality pepper. Good quality pepper seeds can improve products and reduce the unnecessary losses caused by defective pepper seeds during the production process, including sowing, growing, and harvesting.

The traditional and most common methods for selecting superior seeds of high quality are based on physical properties, such as weight, as well as germination, biochemical, and physical tests [1]. Germination tests require 8–14 days to complete the evaluation. Tetrazolium or electrical conductivity tests can also be used to evaluate seed viability rapidly, but they require specialized training and experience for sample preparation and the use of chemicals. Thus, nondestructive methods for assessing seed viability are required by farmers and workers in the seed industry. Near-infrared (NIR) spectroscopy [2,3], Fourier transform NIR [4], and Raman spectroscopy [5–7] have been used to measure the composition of seeds and to estimate seed viability. These spectroscopic techniques are used to determine the concentrations of specific compounds in a sample by analyzing the average spectrum obtained from part of a sample. The typical spectrum might represent a diluted part of the overall sample information. Imaging techniques can obtain image data from the sample information. Various nondestructive imaging techniques have been investigated for the rapid inspection of food and agricultural products with quality and safety applications. In particular, hyperspectral imaging is a promising nondestructive estimation technique that combines spectroscopy and imaging technology [8]. Research into the applications of hyperspectral imaging in the field of food and agriculture have increased recently, particularly defect detection in apples [9,10] and tomatoes [11], as well as the assessment of bacterial biofilms [12]. Hyperspectral imaging acquires spatial and spectral information simultaneously for each pixel in the sample images. This technique can also be used to determine the subtle physical and chemical characteristics of an object, and to visualize the chemical image mapping of the spatial distributions of the chemical components. In addition, research has been conducted into multispectral and hyperspectral imaging techniques that use LED illumination instead of tungsten-halogen illumination to produce high level outputs of blue, green, and red wavelengths, as well as a longer bulb life and less heat [13,14].

The objective of the present study was to develop a partial least square regression-discrimination (PLS-DA) algorithm to identify viable pepper seeds with high germination potential. In addition, the optimal LED illumination conditions were determined and used to develop an algorithm for seed discrimination based on hyperspectral imaging.

## 2. Experimental Section

### 2.1. Materials

Pepper seeds without any coating treatments were purchased from a local seller (Jeil Seed Inc., Suwon, Korea) in 2012. To assess the germination characteristics with aging, 300 seeds were kept in a chamber with a constant relative humidity of 96% and a temperature of 45 °C for 18 days to artificially accelerate the aging process. The seeds were then dried at 22 °C for 24 h to ensure that they had identical moisture contents. In total, 300 normal seeds and 300 aged seeds were vacuum packed and stored at 4 °C. The seeds were allowed to equilibrate to room temperature before hyperspectral imaging and germination.

### 2.2. Hyperspectral Imaging System

**Figure 1.**
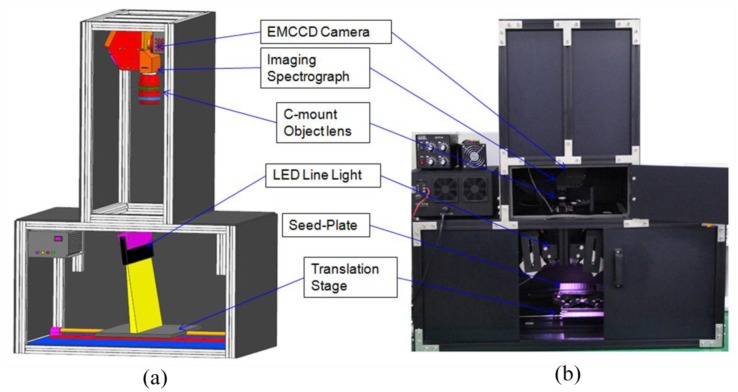
(**a**) Schematic diagram and (**b**) photograph of the hyperspectral reflectance imaging system.

The line-scan hyperspectral imaging system shown in Figure 1 comprised a hyperspectral image measurement unit, illumination source, sample translation unit, and data acquisition unit. The hyperspectral image measurement unit had three components: a low-light sensitive electron multiplying charge-coupled device (EMCCD) camera (MegaLuca R, ANDOR Technology, South Windsor, CT, USA) with 1,004 × 1,002 pixels, an imaging spectrograph (VNIR, Headwall Photonics, Fitchburg, MA, USA) that produced a spectrum for each wavelength between 400 and 1,000 nm, and a C-mount object lens (F1.9, 24 mm; Schneider Optics, Hauppauge, NY, USA) with a 25-mm focal length.

The hyperspectral image measurement unit had a resolution of 8 × 8 μm and was thermoelectrically cooled to −20 °C via a two-stage Peltier device. The EMCCD captured 14-bit images at a rate of 12.5 MHz. Three types of light emitting diodes (LEDs; HPLS-RGB, LVS Co. Ltd, Incheon, Korea), *i.e.*, a blue LED (BL) at 3.6 W with a peak wavelength of 462 nm, a green LED (GL) at 6.45 W with a peak wavelength of 525 nm, and a red LED (RL) at 5.85 W with a peak wavelength of 625 nm, were used to generate the line-scan imaging system's illumination during reflectance imaging. These LED lights had high-level outputs at blue, green, and red wavelengths, which improved the resolution of the seed signals in these wavelength ranges when used to evaluate the pepper seeds, as compared with halogen lights. The output of each LED illumination source was controlled separately between 0 and 100%. A circular optical fiber cable was connected to the light source and a line-shaped optical fiber cable was fixed perpendicular to the direction of transfer at an angle of 15 degrees. Light was emitted from the optical fibers in the shape of a thin line. The reflected light, which passed through the samples, the camera lens, and a 25 μm × 18 mm (width × length) aperture slit, was separated into wavelengths by the imaging spectrograph. The spectra image of the amplified separated light was stored by the EMCCD camera. The sample translation unit comprised a translation stage, step motor (XN10-0180-m02-21, Velmex, New York, NY, USA), and step motor drive. The movement of a sample fixed to the translation stage was controlled by the step interval and the number of steps.

### 2.3. Hyperspectral Image Spectra Acquisition

The sample seeds were arranged in a 10 × 10 grid and fixed onto the translation stage. Hyperspectral images of the top and bottom of all 600 samples were captured to obtain a total of 1,200 images. The images were acquired by line scanning using the three LED illumination sources. The EMCCD exposure time, step sizes, and the number of line scans were 2 ms, 0.1 mm, and 800, respectively. The vertical distance between the samples and lens was 315 mm. The hyperspectral images comprised 640 × 800 pixels, each with 125 wavebands in the range of 400–1,000 nm.

**Figure 2.**
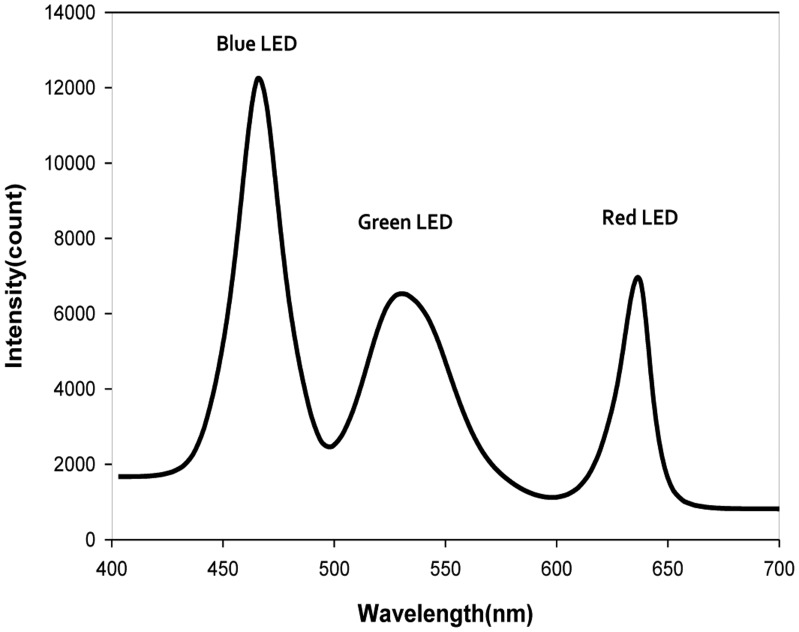
Spectra of the reference panel with blue, green, and red LED illumination.

To investigate the stability of the illumination source, the reflectance spectra of a white Teflon plate with a reflectivity of 99% were measured hourly where the illumination power conditions of the BL, GL, and RL were 100%, 100%, and 15%, respectively. The reflectance spectra of BL, GL, and RL were in the 400–500 nm, 500–600 nm, and 600–700 nm regions, respectively, as shown in Figure 2. All three types of illumination source stabilized within 20 min after activation and the variation in the peak after stabilization was less than ±1.5%. The hyperspectral images were obtained after the illumination sources stabilized.

### 2.4. Germination Test

After obtaining the hyperspectral reflectance images, a germination test was performed according to the guidelines prescribed by the International Seed Testing Association [1] to discriminate the viable and nonviable seeds. Twenty seeds were arranged at regular intervals (5 × 4) on wet paper. The wet paper was rolled, put into a plastic container, and placed on the middle shelf of a chamber with a relative humidity of 65% and a constant temperature of 25 °C. After eight days, the seeds with an emerged radicle that measured ≥1 mm in length were considered to have germinated.

### 2.5. Pretreatment of Hyperspectral Image Data

The hyperspectral images of seeds were transformed into hyperspectral reflectance images using Equation (1) in order to remove the noise generated by the device and the effects of uneven light source intensities. Hyperspectral images of a dark reference were obtained without any light source in order to determine the device noise. Hyperspectral images of the white reference, *i.e.*, a Teflon plate that produced reflectivity of > 99%, were obtained to calibrate the intensity of the light source at each vertical pixel:

(1) $${\text{I}}_{\text{reflectance}}(\text{i})(\%)=\frac{{\text{I}}_{\text{raw}}(\text{i})-{\text{I}}_{\text{dark}}}{{\text{I}}_{\text{white}}-{\text{I}}_{\text{dark}}}\times 100$$

where *I_reflectance_* is the corrected reflectance image at the *i^th^* wavelength, *I_raw_* is the raw hyperspectral image at the *i^th^* wavelength, *I_dark_* is the hyperspectral image of the dark reference at the *i^th^* wavelength, and *I_white_* is the hyperspectral image of the white reference at the *i^th^* wavelength. The sample holder was removed from the transformed hyperspectral reflectance images, and the seed portion of each hyperspectral reflectance spectrum was extracted. There were 173–325 spectra for each seed, thus the spectra were averaged before further analysis.

### 2.6. Prediction of Germination Using Hyperspectral Reflectance Spectra

A viability prediction model was developed using PLS-DA and the classification rate (%) of this model was evaluated. PLS-DA models were developed for the three different types of LEDs (blue, green, and red) and a combination of the three LEDs, *i.e.*, RGB LED, using the preprocessed spectra and they were compared to determine the optimal pretreatment and optimal wavelength range for determining seed viability. Several preprocessing techniques, *i.e.*, smoothing, mean normalization, maximum normalization, range normalization, first-order derivative, second-order derivative, standard normal variate (SNV), and multiplicative scattering correction (MSC) techniques, were applied to the measured spectra to reduce the systematic noise and variation produced by the light sources, and the effects of light scattering due to the seed surface. The 1,200 spectra obtained from the average spectrum of the total pixels that constituted each seed image were divided into two groups to develop the PLS-DA model. We used 840 randomly selected spectra to develop the model and 360 spectra for testing the model. The PLS-DA was used to separate the seeds into two groups based on the spectra set [15]. The model categorized the spectra as either ‘0’ or ‘2’ where ‘0’ indicated nonviable seeds and ‘2’ represented viable seeds.

A cross-validation method was applied to the PLS-DA models and the accuracy of each model was represented by its coefficient of determination for calibration (R_c_^2^), root mean squared error for calibration (RMSEC), coefficient of determination for validation (R_v_^2^), root mean squared error for prediction (RMSEP), and optimal factor (F).

### 2.7. Predicting Germination Using the Hyperspectral Reflectance Images

As shown in Equation (2), the PLS images comprised estimated values, which were obtained by applying regression coefficients, *i.e.*, the weights for each wavelength in the PLS-DA model, to the spectra of each pixel:

(2) $$\text{PLS Image}=\sum_{\text{i}=1}^{\text{n}}{\text{I}}_{\text{i}}{\text{R}}_{\text{i}}+\text{C}$$

where *I_i_* is the *i_th_* image of n spectral images, *R_i_* is the regression coefficient derived from the PLS-DA model, and *C* is the constant of the PLS-DA model.

MATLAB (Version 7.0.4, The Mathworks, Natick, MA, USA) was used to extract and analyze the hyperspectral image data and Unscrambler v 9.2 (CAMO Co., Oslo, Norway) was used to develop and evaluate the PLS-DA models.

## 3. Results and Discussion

### 3.1. Germination Test

To identify the characteristics of aged pepper seeds, a germination test was performed using 20 healthy seeds and 20 artificially aged seeds. The germination rate was > 90% for seeds aged <14 days but it decreased with further aging treatment. Viability was lost by the 18th day of the aging treatment. Therefore, we used 300 normal seeds and 300 seeds that had been aged for 18 days to develop our model, where the viability was predicted from hyperspectral images. The overall germination rate for healthy and aged seeds was 50.5%.

### 3.2. Spectral Characteristics of Pepper Seeds

**Figure 3.**
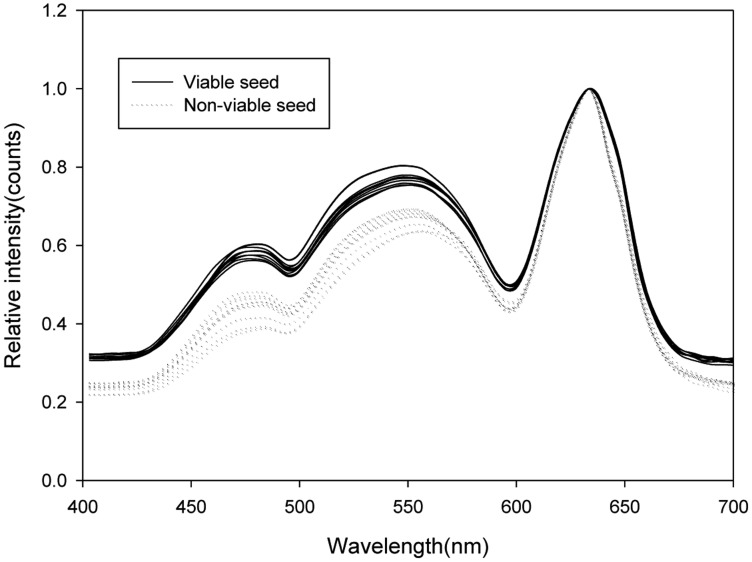
Average spectra with pretreated maximum normalization extracted from images of viable and nonviable pepper seeds illuminated by BL, GL, and RL.

Figure 3 shows the average spectra for pixels with maximum normalization pretreatment at 633 nm, which were extracted from images of 10 viable and 10 nonviable seeds. The reflectance spectra of the seeds are shown for the following wavelength ranges: 400–500 nm (BL), 500–600 nm (GL), and 600–700 nm (RL). The intensities of the spectra of the viable seeds were higher than those of the nonviable seeds in the range of 400–700 nm.

The absorption wavelengths of chlorophyll a and chlorophyll *b*, *i.e.*, 410 nm, 430 nm, 453 nm, 642 nm, and 665 nm, were included in the wavelength ranges of the BL and RL. The viable seeds had larger spectrum peaks in the wavelength range of the BL than the nonviable seeds because the low chlorophyll fluorescence increased the intensity of the spectrum peak for the viable seeds [16]. Thus, low chlorophyll fluorescence was associated with an increased germination rate [17]. Therefore, the germination quality could be predicted using the spectral reflectance from the three types of LED illumination.

### 3.3. Germination Quality Prediction Model Using Hyperspectral Reflectance Spectra

A PLS-DA model was developed to discriminate viable seeds from nonviable seeds using the hyperspectral reflectance spectra obtained after the pretreatments. We investigated the optimal pretreatments of the spectra to enhance the classification performance of the PLS-DA model. Estimated values that exceeded the classification value were considered to be viable seeds. Otherwise, they were considered to be nonviable seeds. The classification value that resulted in the minimum error was selected for use in the model.

#### 3.3.1. Germination Quality Prediction Model Using BL Reflectance Spectra

**Figure 4.**
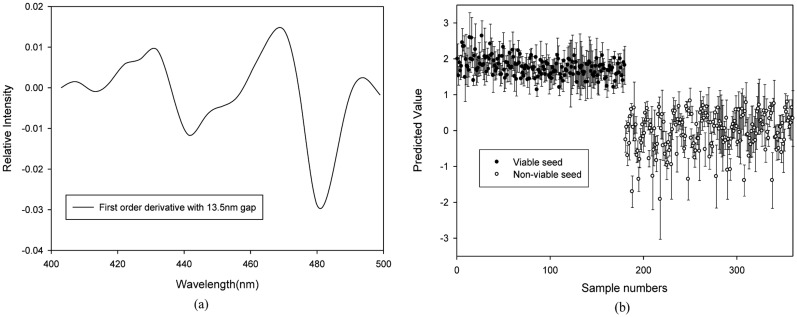
(**a**) Regression coefficients plot of the PLS-DA model, and (**b**) the PLS-DA prediction results obtained using the first-order derivative with a gap of 13.5 nm to discriminate between viable and nonviable pepper seeds based on the reflectance spectra produced under BL (400–500 nm).

The results of calibration, validation, and prediction for the PLS-DA models using various pretreatments to discriminate the seed viability under blue LED illumination are shown in Table 1. The R_v_^2^ and SEP for the PLS-DA model developed using the raw spectra performed better than the other models with pretreatments. However, the best prediction of the pepper seed viability using the PLS-DA models was obtained with the model pretreated by the first-order derivative with a 13.5-nm spectral interval. The optimal number of factors for this model was five. Using a classification value (CV) of 0.974, the model had a discrimination accuracy of 89.4% for viable seeds and 95.0% for nonviable seeds (Figure 4a). The regression coefficient plot for this model, which is shown in Figure 4, indicates the presence of some important absorption peaks. The absorption bands at 431.8 nm, 441.7 nm, 470.2 nm, and 479.8 nm are related to chlorophyll *a* [18], lycopene [19], lutein, and β-carotene [20], respectively. Seed carotenoids, including lutein and β-carotene, are accumulated in seeds during accelerated aging [20] and the high levels of lycopene in tomato seeds contribute to the delayed seed germination caused by higher levels of abscisic acid [21].

#### 3.3.2. Germination Quality Prediction Model Using GL Reflectance Spectra

**Figure 5.**
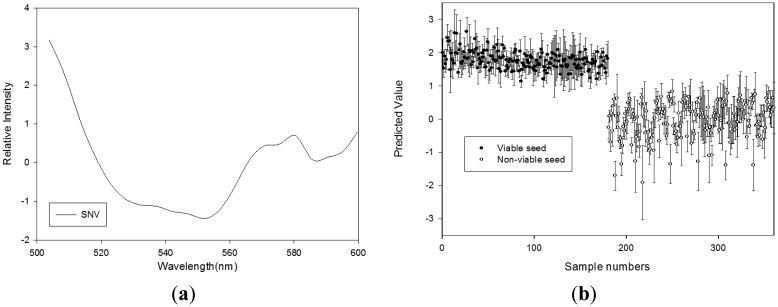
(**a**) Regression coefficients of the PLS-DA model, and (**b**) the PLS-DA prediction results obtained using SNV to discriminate between viable and nonviable pepper seeds based on the reflectance spectra produced under GL (500–600 nm).

Table 2 shows the results obtained using the PLS-DA models with various pretreatments to discriminate the seed viability under GL illumination. The PLS-DA models that used the SNV, range normalization, and first-order derivative for calibration and validation produced better results than the non-pretreated PLS-DA model. The optimal pretreatment for the PLS-DA model in terms of calibration, validation, and prediction was the SNV method. The value of the PLS factor was 3 and the regression coefficients with this model are shown in Figure 5a. The high regression coefficient represented the absorption bands at 580 nm, which were related to chlorophyll *a* [18]. The R_c_^2^ value was 0.897 and SEC was 0.359 for the calibration of this model. The cross-validation yielded an R_V_^2^ of 0.870 and SEP of 0.361. Figure 5b shows the prediction results obtained using a classification value of 0.786, where the discrimination accuracies for viable seeds and nonviable seeds were 85% and 97.2%, respectively.

#### 3.3.3. Germination Quality Prediction Model Using RL Reflectance Spectra

**Figure 6.**
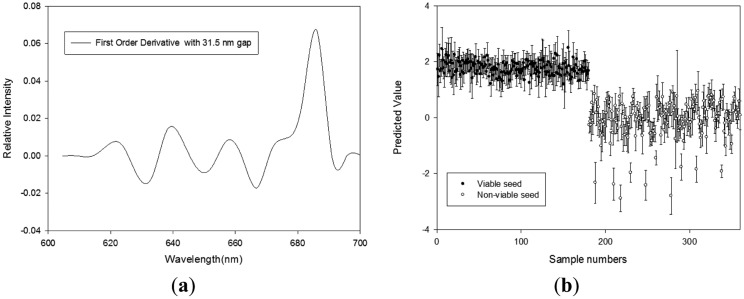
(**a**) Regression coefficients of the PLS-DA model, and (**b**) the PLS-DA prediction results obtained using the first-order derivative with a 31.5 nm interval to discriminate between viable and nonviable pepper seeds based on the reflectance spectra under RL (600–700 nm).

Table 3 shows the results obtained using the PLS-DA models with various pretreatments to discriminate the seed viability under RL illumination. The results of the PLS-DA models used for calibration, validation, and prediction were improved by smoothing and the first-order derivative pretreatment. The best PLS-DA results in terms of seed viability discrimination were obtained using the first derivative spectral pretreatment with a 31.5 nm spectral interval and a six-factor model. The R_c_^2^ and SEC values with this model were 0.918 and 0.2865, respectively, for calibration. The R_v_^2^ and SEP were 0.9165 and 0.2895, respectively, for validation. The CV of this model was 0.995 and the discrimination accuracies for viable seeds and nonviable seeds were 88.9% and 90.0%, respectively (Figure 6c). The higher regression coefficients at 639.4 nm, 666.9 nm, and 672 nm with this model, which are shown in Figure 6a, represent the characteristics of chlorophyll *a* and chlorophyll *b* [18].

#### 3.3.4. Germination Quality Prediction Model Using RGB LED Reflectance Spectra

**Figure 7.**
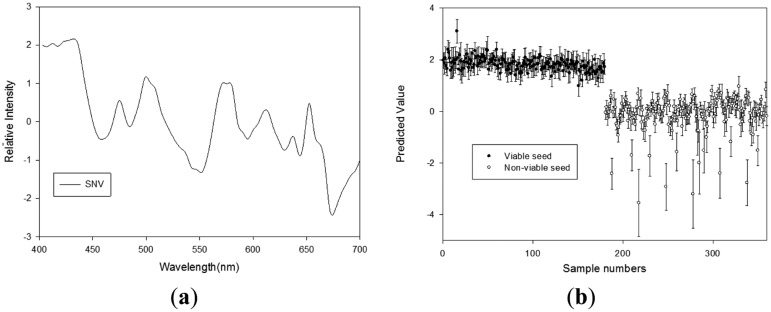
(**a**) Regression coefficients of the PLS-DA model, and (**b**) the PLS-DA prediction results obtained using SNV to discriminate between viable and nonviable pepper seeds based on the reflectance spectra under RGB LED (400–700 nm).

The results obtained using the PLS-DA models with various pretreatments to discriminate the seed viability under RGB LED illumination are shown in Table 4. The results of the PLS-DA models used for calibration, validation, and prediction were improved by the pretreatments, except the second-order derivative method. The best results were obtained with the SNV-pretreated spectra, which had the optimal number of three factors. The calibration model had an R_c_^2^ of 0.907 and an SEC of 0.305. The R_v_^2^ and SEP values for the validation model were 0.904 and 0.310, respectively. Figure 6b shows the predictions obtained using this model with a CV of 0.942. The discrimination accuracies of the model with viable and nonviable seeds were 96.7% and 99.4%, respectively. The absorption bands at 434 and 664 nm (chlorophyll *a*), 457 and 644 nm (chlorophyll *b*), 672 nm (chlorophyll), 475 nm (lycopene), and 483 nm (β-carotene) had high regression coefficients, as shown in Figure 7a [18,19]. Among the different models we developed, the PLS-DA model that used the RGB LED illumination reflectance spectra had the highest discrimination accuracy, and chlorophyll and carotenoids in the range of 400–700 nm may perform better than other ranges for discriminating viable seeds [17,20].

### 3.4. Determination of the Germination Quality Using Hyperspectral Reflectance Images

**Figure 8.**
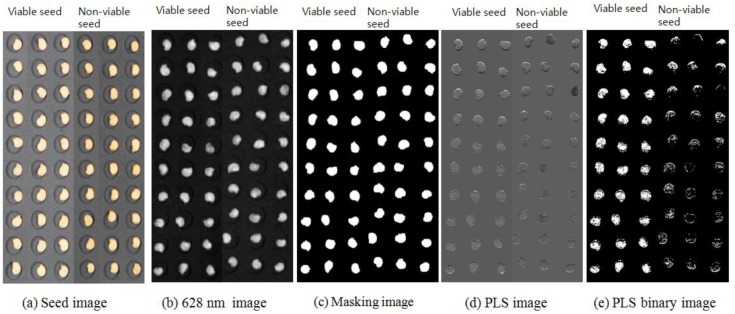
PLS image processing to classify viable and nonviable pepper seeds.

Hyperspectral data processing was performed to determine the germination quality using the hyperspectral image data with pretreatment, where the highest discrimination accuracies were evaluated with BL, GL, RL, and RGB LED illumination. As shown in Figure 8, the 599.8 nm images obtained from seeds with no pretreatment were converted into binary images, where values greater than the threshold value 0.25 were converted to ‘1’ and those less than the threshold value were converted to ‘0’. The backgrounds were then erased and a masking image was produced for the seeds only. PLS images were created by applying regression coefficients from the PLSR model with pretreatment to the hyperspectral images with the same pretreatment, and then applying the masking image. The PLS images were converted into binary images using the CV of the PLS-DA model with SNV pretreatment for the RGB LED reflectance spectra in Table 4 (0.942) as the threshold value of the PLS image. The binary PLS images represented the vigorous parts of seeds.

**Figure 9.**
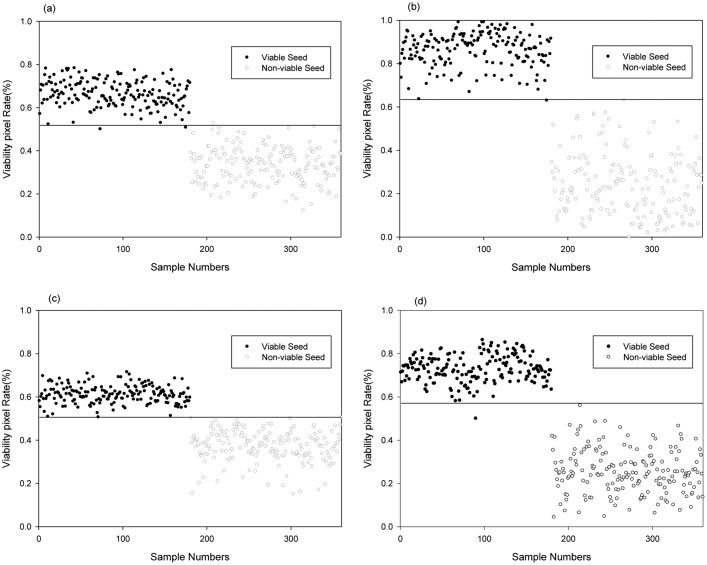
PLS image prediction results obtained when discriminating between viable and nonviable pepper seeds using spectral images of seeds illuminated by: (**a**) BL (400–500 nm), (**b**) GL (500–600 nm), (**c**) RL (600–700 nm), and (**d**) a RGB LED (400–700 nm), where the PLS-DA was applied.

Table 5 shows the discrimination results obtained for viable and nonviable pepper seeds using the PLS images and the optimal PLS-DA model for each LED illumination type. In total, 86,882 spectra from 360 seed images were used to verify the optimal PLS-DA model with four types of LED illumination spectra (Figure 9). Figure 8e shows the binary PLS images of seeds, where the vigorous parts of each seed comprise pixels with an image value of 1. There were 183–310 pixel spectra per seed. The best results predicted for the PLS images used the PLS-DA model with the first-order derivative based on the RL illumination spectra. In this model, the seed was considered to be viable if > 50.6% of the pixels for a given seed had an image value of 1. The seed was considered to be nonviable if < 50.6% of the pixels had an image value of 1. A threshold value of 50.6% was found to yield the best discrimination accuracy. The discrimination accuracies for 180 viable seeds and 180 nonviable seeds were 100% and 100%, respectively (Figure 9). The discrimination accuracies for viable seeds and nonviable seeds were 98.9% and 99.4% with BL illumination, 99.4% and 100% with GL illumination, and 99.4% and 100% with RGB LED illumination, respectively. The pixel rates of viable seeds were distributed in a narrow range of 50.62%–71.49% with the PLS-DA model using the first-order derivative in the range of RL illumination, but in a wider range of 50.0%–86.31% with the PLS-DA model that used the SNV in the range of RGB illumination. Thus, the discrimination accuracy of the model based on RL illumination was higher than that of the model based on RGB illumination.

The PLS image method that discriminated the viable seeds based on the ratio of vigorous pixels with an image value of 1, which were predicted for each spectrum pixel of a seed using the PLS-DA model, was more accurate than the method that discriminated viable seed using a representative spectrum of averaged spectrum pixels of a seed using the same PLS-DA model. Thus, the hyperspectral imaging technique is more appropriate for the determination of seed viability than conventional spectroscopy using an averaged spectrum.

## 4. Conclusions

The aim of this study was to develop a new technique for discriminating between viable and nonviable pepper seeds. A PLS-DA model was developed using hyperspectral images of pepper seeds generated under LED illumination. In addition, an algorithm for hyperspectral image processing was developed using the optimal PLS-DA model.

The PLS-DA model developed using the hyperspectral reflectance image spectra with the SNV based on RGB LED illumination (range of 400–700 nm) yielded discrimination accuracies of 96.7% and 99.4% for viable seeds and nonviable seeds, respectively. Illumination with RGB LED facilitated the best discrimination between viable and nonviable seeds. The hyperspectral reflectance images successfully identified aged, nonviable seeds.

Processing hyperspectral images using the PLS-DA model with the first-order derivative of the 31.5-nm gap under RL illumination (in the range of 600–700 nm) yielded 100% discrimination accuracy with both viable and nonviable seeds. Thus, hyperspectral reflectance imaging has considerable potential for identifying superior seeds among large volumes of seeds.

## Figures and Tables

**Table 1.** Results obtained using the PLS-DA models based on the reflectance spectra with BL illumination (400–500 nm).

	**Calibration**		**Validation**		**F^a^**	**Prediction**		
	
	**R_c_^2^**	**RMSEC**	**R_v_^2^**	**RMSEP**		**CCR^b^**		**CV^e^**

						**VS^c^**	**NVS^d^**	
Non-pretreatment	0.860	0.374	0.854	0.383	7	90.6%	89.4%	0.899
Mean Normalization	0.845	0.393	0.839	0.401	6	82.8%	93.3%	0.866
Maximum Normalization	0.855	0.380	0.849	0.389	7	92.8%	85.6%	0.995
Range Normalization	0.853	0.383	0.847	0.392	7	86.7%	91.1%	0.912
SNV	0.848	0.390	0.842	0.398	5	91.1%	91.1%	0.898
MSC	0.834	0.407	0.828	0.416	5	85.6%	91.1%	0.944
Smoothing	0.856	0.379	0.851	0.387	8	89.4%	88.9%	0.88
**1^st^ order Derivative**	**0.853**	**0.384**	**0.849**	**0.389**	**5**	**89.4%**	**95.0%**	**0.974**
2^nd^ order Derivative	0.856	0.379	0.851	0.387	7	89.4%	92.2%	0.850

Notes:

a F, number of factors;

b CCR, correct classification rate;

c VS, viable seed;

d NVS, non-viable seed;

e CV, classification value.

**Table 2.** Results obtained using the PLS-DA models based on the reflectance spectra with GL illumination (500–600 nm).

	**Calibration**		**Validation**		**F^a^**	**Prediction**		
	
	**R_c_^2^**	**RMSEC**	**R_v_^2^**	**RMSEP**		**CCR^b^**		**CV^e^**

						**VS^c^**	**NVS^d^**	
Non-pretreatment	0.847	0.391	0.845	0.394	4	88.9%	94.4%	0.987
Mean Normalization	0.848	0.391	0.844	0.395	4	76.7%	95.6%	0.873
Maximum Normalization	0.849	0.389	0.845	0.394	4	88.3%	92.2%	0.952
Range Normalization	0.868	0.363	0.866	0.367	4	88.3%	89.4%	1.011
**SNV**	**0.897**	**0.359**	**0.870**	**0.361**	**3**	**85.0%**	**97.2%**	**0.786**
MSC	0.832	0.410	0.829	0.414	3	86.7%	91.7%	0.971
Smoothing	0.845	0.394	0.843	0.397	4	93.3%	90.0%	1.056
1^st^ order Derivative	0.848	0.389	0.847	0.392	3	89.4%	95.0%	0.974
2^nd^ order Derivative	0.702	0.546	0.692	0.553	5	41.1%	78.9%	0.758

Notes:

a F, number of factors;

b CCR, correct classification rate;

c VS, viable seed;

d NVS, non-viable seed;

e CV, classification value.

**Table 3.** Results obtained using the PLS-DA models based on the reflectance spectra with RL illumination (600–700 nm).

	**Calibration**		**Validation**		**F^a^**	**Prediction**		
	
	**R_c_^2^**	**RMSEC**	**R_v_^2^**	**RMSEP**		**CCR ^b^**		**CV ^e^**

						**VS^c^**	**NVS^d^**	
Non-pretreatment	0.835	0.407	0.830	0.413	7	89.4%	82.8%	1.061
Mean Normalization	0.820	0.424	0.815	0.430	6	86.7%	89.4%	0.967
Maximum Normalization	0.821	0.423	0.817	0.428	6	88.9%	82.8%	1.028
Range Normalization	0.822	0.422	0.819	0.426	5	86.7%	81.1%	1.014
SNV	0.826	0.417	0.822	0.422	5	85.0%	87.8%	0.96
MSC	0.808	0.438	0.803	0.445	5	80.0%	82.8%	0.972
Smoothing	0.846	0.392	0.844	0.396	6	90.0%	89.4%	0.997
**1^st^ order Derivative**	**0.847**	**0.391**	**0.844**	**0.396**	**6**	**88.9%**	**90.0%**	**0.995**
2^nd^ order Derivative	0.709	0.539	0.697	0.551	10	57.2%	70.0%	0.83

Notes:

a F, number of factors;

b CCR, correct classification rate;

c VS, viable seed;

d NVS, non-viable seed;

e CV, classification value.

**Table 4.** Result obtained using the PLS-DA models based on reflectance spectra with RGB LED illumination (400–700 nm).

	**Calibration**		**Validation**		**F^a^**	**Prediction**		
	
	**R_c_^2^**	**RMSEC**	**R_v_^2^**	**RMSEP**		**CCR^b^**		**CV^e^**

						**VS^c^**	**NVS^d^**	
Non-pretreatment	0.887	0.337	0.883	0.342	6	95.0%	95.6%	1.01
Mean Normalization	0.888	0.334	0.885	0.339	6	96.1%	97.8%	1.014
Maximum Normalization	0.894	0.326	0.891	0.331	6	95.6%	97.2%	1.041
Range Normalization	0.894	0.326	0.891	0.331	6	96.7%	95.0%	1.106
**SNV**	**0.907**	**0.305**	**0.904**	**0.310**	**5**	**96.7%**	**99.4%**	**0.942**
MSC	0.899	0.317	0.897	0.322	5	97.2%	98.3%	0.985
Smoothing	0.893	0.327	0.890	0.332	7	95.6%	96.1%	1.063
1^st^ order Derivative	0.889	0.333	0.885	0.340	7	96.7%	96.7%	1.05
2^nd^ order Derivative	0.892	0.329	0.880	0.355	8	90.0%	92.2%	0.955

Notes:

a F, number of factors;

b CCR, correct classification rate;

c VS, viable seed;

d NVS, non-viable seed;

e CV, classification value.

**Table 5.** Discrimination between viable and nonviable pepper seeds using PLS images and the PLS-DA model.

**Type of Illumination**	**Pretreatment**		**Number of Seed**	**Prediction**		

				**Viable Seed**	**Non-Viable Seed**	**Accuracy**
BL (400–500 nm)	1st order derivative with 13.5 nm gap	Viable Seed	180	178	2	98.9%
		Non-Viable Seed	180	1	179	99.4%

GL (500–600 nm)	SNV	Viable Seed	180	179	1	99.4%
		Non-Viable Seed	180	0	180	100%

RL (600–700 nm)	1st order derivative with 31.5 nm gap	Viable Seed	180	180	0	100%
		Non-Viable Seed	180	0	180	100%

RGB LED (400–700 nm)	SNV	Viable Seed	180	179	1	99.4%
		Non-Viable Seed	180	0	180	100%

## References

[1] (2008). International Rules for Seed Testing.

[2] Min T.G., Kang W.S. (2003). Nondestructive separation of viable and non-viable gourd (Lagenaria siceraria) seeds using single seed near infrared reflectance spectroscopy. Korean Soc. Hortic. Sci..

[3] Shetty N., Min T.G., Olesen M.H., Boelt B. (2011). Optimal sample size for predicting viability of cabbage and radish seeds based on near infrared spectra of single seeds. J. Near Infrared Spectrosc..

[4] Lohumi S., Mo C., Kang J.S., Hong S.J., Cho B.K. (2013). Nondestructive evaluation for the viability of watermelon (*citrullus lanatus*) seeds using fourier transform near infrared spectroscopy. J. Biosyst. Eng..

[5] Mo C., Kang S., Lee K., Kim G., Cho B.K., Lim J.G., Lee H., Park J. (2012). Germination prediction of cucumber(*cucumis sativus*) seed using raman spectroscopy. J. Biosyst. Eng..

[6] Reitzenstein S., Rösch P., Strehle M.A., Berg D., Baranska M., Schulz H., Rudloff E., Popp J. (2007). Nondestructive analysis of single rapeseeds by means of Raman spectroscopy. J. Raman Spectrosc..

[7] de Silva C.E., Vandenabeele P., Edwards H.G., de Oliveira L.F. (2008). NIR-FT-Raman spectroscopic analytical characterization of the fruits, seeds, and phytotherapeutic oils from rosehips. Anal. Bioanal. Chem..

[8] Kim M.S., Chen Y.R., Mehl P.M. (2001). Hyperspectral reflectance and fluorescence imaging system for food quality and safety. Trans. Am. Soc. Agric. Eng..

[9] Lee K.J., Kang S.W., Delwiche S.R., Kim M.S., Noh S.H. (2008). Correlation analysis of hyperspectral imagery for multispectral wavelength selection for detection of defects on apples. Sens. Instrum. Food Qual. Saf..

[10] Cho B.K., Beak I.S., Lee N.G., MO C.H. (2011). Study on bruise detection of ‘Fuji’ apple using hyperspectral reflectance imagery. J. Biosyst. Eng..

[11] Jeong D.H., Moon S., Kim M.S., Hoonsoo Lee H., Lee H., Cho B.K. (2013). Detection Algorithm for Cracks on the Surface of Tomatoes using Multispectral Vis/NIR Reflectance Imagery. J. Biosyst. Eng..

[12] Jun W., Kim M.S., Lee K., Millner P., Chao K. (2009). Assessment of bacterial biofilm on stainless steel by hyperspectral fluorescence imaging. Sens. Instrum. Food Qual. Saf..

[13] Lawrence K.C., Park B., Heitschmidt W.R., Windham G.W., Thai C.N. (2007). Evaluation of LED and tungsten-halogen lighting for fecal contaminant detection. Appl. Eng. Agric..

[14] Qin J., Chao K., Kim M.S., Kang S., Jun W. (2011). Detection of organic residues on poultry processing equipment surfaces by LED-induced fluorescence imaging. Appl. Eng. Agric..

[15] Alexandrakis D., Downey G., Scannell A.G.M. (2008). Detection and identification of bacteriain an isolated system with near-infrared spectroscopy and multivariate analysis. J. Agric. Food Chem..

[16] Merzlyak M.N., Solovchenko A.E., Gitelson A.A. (2003). Reflectance spectral features and non-destructive estimation of chlorophyll, carotenoid and anthocyanin content in apple fruit. Postharvest Biol. Technol..

[17] Jalink H., Scoor R.V., Frandas A., Pijlen J.G., Raoul J.B. (1998). Chlorophyll fluorescence of Brassica oleracea seeds as a non-destructive marker for seed maturity and seed performance. J. Seed Sci. Res..

[18] Gross J. (1991). Pigments in Vegetables: Chlorophylls and Carotenoids.

[19] Elmier S.M., Mackinney G., Zscheile F.P. (1935). Absorption spectra of alpha and beta carotenes and lycopene. Plant Physiol..

[20] Smolikova G., Laman N., Boriskevich O. (2011). Role of chlorophylls and carotenoids in seed tolerance to abiotic stressors. Russian J. Plant Physiol..

[21] Ramirez-Rosales G., Bennett M., McDonald M., Francis D. (2004). Effect of fruit development on the germination and vigor of high lycopene tomato (*Lycopersicon esculentum Mill.*) seeds. Seed Sci. Technol..

